# Supplementary material for: Are carbon emissions trading and green financial instruments synergistic? -Comprehensive quantitative research based on content analysis
Source: PLoS One. 2024 Mar 7;19(3):e0298601. doi: 10.1371/journal.pone.0298601 (PMC10919601; doi:10.1371/journal.pone.0298601)
Supplement: S1 Data — (ZIP) [file pone.0298601.s001.zip › Data/地级市Z_F工作报告.zip!/╜¡╬≈/╥╦┤║╩╨/└┤╫╘╣1⁄2╓┌║┼í╛┬φ┐╦╩2╛▌═°í┐_2008.docx]

各位代表：

现在，我代表市人民政府向大会作政府工作报告，请予审议，并请各位市政协委员和列席会议的同志提出意见。

　　一、2007年工作简要回顾

　　2007年，是全面贯彻市第二次党代会精神的第一年，也是本届政府全面履职的第一年。一年来，全市人民在省委、省政府和市委的正确领导下，认真落实科学发展观，深入实施“心圈廊”发展战略，全面打响中心城“三大战役”，加快推进“四个宜春”建设，市二届人大二次会议确定的各项目标全面完成。预计完成生产总值512亿元，比上年增长14%；财政总收入50.32亿元，其中地方财政收入28.36亿元，分别增长30.1%和29.9%；规模以上工业增加值148亿元，增长28%；全社会固定资产投资253.6亿元，增长25.1%；社会消费品零售总额154.6亿元，增长17.6%；城镇居民人均可支配收入1.1万元，农民人均纯收入4020元，分别增长20.2%和10.1%，居民消费价格总水平上涨4个百分点，城镇登记失业率4.2%；人口自然增长率控制在7.9‰以内。在建设和谐小康宜春的进程中，我们又迈出了坚实的一步。

　　加快结构调整和发展方式转变，经济增长的质量和效益进一步提高。经济结构继续优化。2007年，三次产业占GDP的比重由上年的23.1：46.6：30.3调整为20.8：50.1：29.1，第二产业首次突破50%份额。财政总收入占GDP的比重达到9.8%，同比提高1个百分点，税收收入占财政总收入的比重达到90.1%，同比提高2.6个百分点。开放型经济有效拓展。签约重大工业及旅游开发项目68个，其中投资5亿元以上的10个。预计实际利用外资2.6亿美元，增长10.2%；引进工业项目资金120亿元，增长25.3%；出口创汇2亿美元，增长33.4%。着力推进宜春医药、奉新纺织、樟树盐化工、丰城煤电、高安建筑陶瓷、上高鞋革“六大产业基地”建设，9个工业园区实现销售收入356亿元，增长38%；实现利税38亿元，增长22%。深入推进全民创业，以民营经济为主体的经济增长格局凸显，预计民营企业纳税28亿元，占据财政收入的“半壁江山”。樟树仁和获中国驰名商标，樟树春丝面条、玉珠大米、金虎保险柜获中国名牌产品称号。资源节约和环境保护得到加强。基本农田保护率稳定在85%以上。争取国家投资土地整理项目7个，总投资1.44亿元。启动了114个“空心村”治理工作。大力推进土地资源市场化配置，规范土地市场。强化环境监管和污染防治，启动了绿色生态宜春建设，开展了工业园区污水治理等八个专项行动。宜丰官山获批国家级自然保护区。饮用水源得到保护。落实节能减排工作任务，丰城工业园列入全省六个循环经济试点园区之一。

　　坚持统筹兼顾，发展的持续性和协调性进一步增强。统筹城乡发展，新农村建设迈出新步伐，城市创建取得重大突破。发放粮食直补、良种补贴等各类涉农补贴资金4.15亿元，粮食生产连续五年增产。农业产业化稳步发展，17家龙头企业、21个生产基地和161个“一村一品”示范村纳入全省“十百千”工程，宜丰县10万亩水稻标准化生产基地被列为“全国安全优质农产品十大基地”，奉新绿色大米农业标准化示范区通过国家级考核验收。全国首家籼稻中远期现货电子交易中心开通营运。造林步伐加快，完成造林面积47.4万亩。城乡人居环境和生产条件继续完善。全市1082个省定新农村建设试点村安装自来水40709户，建造水冲厕29617个，改造通村组道路730.47公里，建设沼气池11300个。战胜了近50年来的特大旱灾，478座病险水库完成除险加固，320国道大万段改造、武吉高速公路、宜安公路建设初步完成，完成市养公路改造160公里，县乡水泥(油)路竣工里程1200公里，新增通水泥(油)路建制村290个。圆满完成了交通沿线村庄建筑环境整治。投入城市建设资金22.1亿元，城镇化率达到36.2%。围绕建设“中部地区最佳宜居城市”目标，中心城启动了“13579”绿色工程建设，创建“中国优秀旅游城市”一次申报、一次摘牌，明月山温泉风景名胜区、三爪仑国家示范森林公园、明月山天沐温泉度假村被评为国家4A级旅游景区，创建“国家卫生城”工作通过全国爱卫办技术评估，创建“全国水土保持生态环境建设示范城市”通过复验，创建全省第二届文明城市工作成效明显，城市管理工作连续五年列全省第一。统筹区域发展，促进心圈廊优势互补、各展所长。“三大战役”取得阶段性成果，宜春经济开发区签约重大项目11个，与上年相比，主营业务收入和财政收入均实现翻番，袁州区新扩建了彬江工业基地；宜阳新区规划基本完成，核心区工程全面拉开；首届月亮文化节暨经贸活动周成功举办，以明月山温泉风景名胜区为龙头的旅游持续升温。“丰樟高经济圈”产业基地聚集效应明显、发展势头强劲，丰城跻身全国县域经济200强，樟树成功承办了第38届全国药交会。320国道工业走廊、山区生态经济走廊产业规模和发展水平不断提升。统筹经济社会发展，社会事业全面进步。加强自主创新能力建设，科技进园入企、进村入户和成果转化工程有力推进。“两基”工作顺利通过“国检”，高安中学在全国中学生物理竞赛中获江西赛区团体特等奖，宜春实验中学校园剧《童心》获全国第二届中小学生艺术展演一等奖，宜春学院顺利通过教育部本科教学工作水平评估。靖安水口东周古墓直接参评2007年中国10大考古新发现，樟树吴城遗址、丰城洪州窑遗址保护与利用规划方案通过省专家组评审，一批优秀戏剧小品在全国获大奖，铜鼓“秋收起义80周年纪念活动”、《中国红歌会—走进上高》、“红歌唱响新宜春”电视歌手大奖赛均成功举办。7个县级疾控中心实验室建设全面完成，11个卫生监督所全部独立运行。成功开展了“群众体育年”系列活动，承办了第14届全国冬泳锦标赛等25项省级以上赛事，宜春籍运动员杨文军、冯桂鑫在国际比赛中夺冠。人口和计划生育工作进一步加强，出生人口得到有效控制。

　　不断深化改革，持续发展的活力和动力进一步提升。规范落实了公务员津贴补贴政策，“乡财县代管”试点乡镇达到143个，深化了市本级部门预算改革，推行了政府收支分类改革，国库集中收付制度改革逐步深入，惠农各项补贴资金全部纳入“一卡通”发放，启动了国有农场税费改革。市直事业单位综合改革顺利推进，出台了《市直差额拨款事业单位养老保险实施办法》，审定了事业单位“三定”方案。全市机关事业单位工资制度改革基本完成。除铜鼓免检外，9个县（市、区）全部通过林权主体改革省级验收。水管体制改革扎实推进。粮食企业改革继续深化。企业改制上市工作取得新进展，江特电机在深圳证券交易所成功上市。

　　有效实施民生工程，和谐社会建设步伐进一步加快。全市各级财政用于民生工程的新增配套资金达2.55亿元。城镇新增就业6.33万人，对“零就业家庭”做到7个工作日内至少解决其一人实现就业；城镇职工参加基本养老保险人数达41.85万人；纳入保障范围的城市低保对象11.6万人、农村低保对象11.44万人；实施城乡医疗救助人数达24.33万人，救助金额达6829万元；“新农合”参合率87.37%；义务教育免学杂费66.23万人，补助寄宿生生活费3.34万人，免费为12.88万人提供教科书。开工建设经济适用房20.48万平方米，受益家庭2342户；廉租房受益家庭1878户，其中租赁补贴676户，实物配租1202户。认真落实安全生产责任制，深化各项专项整治，安全生产态势平稳。全面开展食品药品安全综合监管体系建设，公众饮食用药安全进一步得到保障。积极畅通信访渠道，大量矛盾纠纷和不稳定因素消除在萌芽状态。深入开展和谐平安创建活动，依法防范和打击违法犯罪活动，有力地维护了社会稳定。

　　狠抓政府自身建设，社会管理和公共服务进一步改善。深入开展“作风建设推进年”活动，在全省民主评议政风行风的52个行业、系统中，我市总评列全省第一名，有28个市直单位荣获全省前三名，其中13个单位获第一名。大力推行政务公开和服务承诺制度，推进电子政务建设，实施市直单位行政审批“两集中、两到位”改革试点工作。坚持向人大报告政府工作，加强与政协联系，认真接受人大及其常委会的法律监督和政协的民主监督，112件人大代表建议、286件政协委员提案全部办复。“五五”普法全面开展，法律援助和法律服务效能继续提升。廉政建设进一步加强，执法监察、廉政监察、效能监察和审计监督成效明显。国防后备力量和民兵预备役建设、人民防空、国家安全、外事侨务、民族宗教、广播电视、涉台事务、气象服务、防震减灾、国有资产管理、妇女儿童、统计、档案、扶贫、扶残、老龄和红十字会等各项工作都取得了新进步。

　　各位代表，回顾过去一年的工作，我们深感成绩的取得，是省委、省政府和市委坚强领导的结果，是市人大依法监督、市政协民主监督以及社会各界大力支持的结果，是全市人民团结一致、共同奋斗的结果。在此，我谨代表市人民政府，向全市广大工人、农民、知识分子和干部，向各民主党派、工商联、无党派人士、各人民团体，向驻宜部队、武警官兵、公安干警、中央和省驻宜单位，致以崇高的敬意！向所有关心、支持宜春发展的离退休老同志和海内外朋友，表示衷心的感谢！

　　我们正处于全面建设小康社会的关键时期，既要面临国际经济环境变化的影响，更要应对中央宏观政策调整以及国内发展竞争加剧带来的挑战与考验，特别是党的十七大胜利召开，科学发展观深入人心，国际国内产业转移和技术扩散加快，国家促进中部地区崛起政策的实施，泛珠三角区域经济合作的展开，给我们带来了难得的发展机遇。我们完全有必要也有条件借助和利用有利的外部发展环境，在经济社会又好又快地发展势头中，实现新的一年“好中求快”。当前，尤其要清醒地认识我市发展中依然存在一些突出矛盾和问题：经济总量与增长速度仍有差距，实现“前移前列”的压力较大；经济结构性矛盾较突出，产业发展的核心竞争力不强，资源环境约束日渐显现，节能减排任务艰巨；城乡和区域发展不平衡，农业抗御自然风险和市场风险的能力仍比较脆弱；社会就业压力依然较大，部分城乡居民生活困难，社会矛盾不断显现，民生保障有待进一步完善；深化改革任重道远，政府职能转变和作风建设必须加强，等等。继续保持和发展我市的好形势，必须立足当前、着眼长远，加快解决经济社会运行中的深层次矛盾。

　　二、2008年工作安排

　　2008年，是全面贯彻党的十七大精神的第一年，也是认真落实市第二次党代会精神、深入推进和谐小康宜春建设的重要一年，做好今年各项工作，对于保持我市良好发展势头，具有十分重要的意义。

　　今年政府工作的总体要求是：全面贯彻落实党的十七大精神，高举中国特色社会主义伟大旗帜，以邓小平理论、“三个代表”重要思想为指导，深入贯彻落实科学发展观，坚持民生为本、开放为先、创新为要、和谐为重、行政为民，紧扣重点工作、重大项目和重要环节，致力做活心圈廊、做强工业园、做大企业群、做旺中心城，不断夯实发展基础，不断壮大经济总量，不断提高人民生活水平，努力实现跨越式发展，全面提升和谐小康宜春建设新水平。

　　具体实践中，必须遵循以下原则：

　　第一，科学发展。致力把加快建设“实力宜春、活力宜春、魅力宜春、和谐宜春”，作为自觉实践科学发展观的集中体现和统一行动，贯穿于宜春在江西崛起中奋勇争先的全过程，立足好中求快，创新发展模式，提高发展质量。

　　第二，以人为本。从满足人民群众日益增长的物质文化需求出发，谋发展、促发展；从人民群众最需要解决的问题入手，办实事、解民忧，妥善处理好各方面的利益关系，推进基本公共服务均等化，努力形成全市人民各尽其能、各得其所、和谐相处的局面。

　　第三，改革创新。充分发挥市场配置资源的基础性作用，加快以市场为取向的各项改革，充分利用国际国内“两个市场、两种资源”，整合一切可以整合的要素，利用一切可以利用的资源，调动一切可以调动的力量，努力构筑充满活力、富有效率、更加开放的体制和格局。

第四，统筹协调。牢固树立和谐发展理念，正确处理改革、发展、稳定的关系，注重发展的系统性、整体性、协调性，统筹推进经济建设、政治建设、文化建设、社会建设和生态建设，不断拓展生产发展、生活富裕、生态文明、社会和谐的发展道路。

　　第五，积极有为。坚持政府工作的项目化、推进的时效性、落实的责任制，根据不同阶段的发展要求，理清工作重点，细化推进措施，明确责任主体，落实完成时限，充分激发各个层面、各个部门和政府全体工作人员的紧迫性、创造性和责任性。

　　综合考虑各种因素，今年国民经济和社会发展主要目标建议为：生产总值增长13%；单位生产总值能耗下降4.4%，主要污染物排放总量削减2%；财政总收入和地方财政收入分别增长20%和18%；规模以上工业增加值增长28%；全社会固定资产投资增长22%；实际利用境外资金增长10%，引进市外工业资金增长11.5%，外贸出口增长10.5%；社会消费品零售总额增长16%；农民人均纯收入增长8%、增加320元，城镇居民人均可支配收入增长8%、增加880元；城镇登记失业率控制在4.5%以内，人口自然增长率控制在7.9‰以内，城镇化率达到38%。这些目标中，节能减排指标是约束性的，只能确保到位；经济增长等指标是预期性的，可以适当调整；富民指标是指令性的，必须完成得更好。

　　为实现上述目标，着重围绕五个方面开展工作：

　　一、咬住重大项目抓突破，增强区域经济发展新活力

　　加速“心圈廊”建设。继续坚持以市场为导向，以利益联结为纽带，以产业整合为重点，积极探索“心”、“圈”、“廊”之间优势互补、共同发展的促进机制，加快建设特色鲜明的区域经济新格局。做大中心城规模、做强“心”经济实力，以宜春经济开发区建设为重点，全力推进工业强攻战，壮大医药、机械电子、新型材料、汽摩配产业，加快发展现代物流业；以宜阳新区建设为重点，打好城市扩张战，拉开城市框架，完善城市基础功能；以明月山景区建设为重点，打好旅游升温战，做响旅游品牌，加快聚集人气、商气和财气。壮大“圈”实力、促进“圈”融合，发展盐化、煤电、建筑陶瓷和精品陶瓷、货运物流等特色产业，促进优势共创、资源共享、协作共进。打造320国道和山区产业经济两“廊”特色，突出发展上高的食品、鞋革加工，奉新的纺织服装，靖安的旅游、有色金属加工，万载、铜鼓、宜丰的山区生态有机走廊。

　　加快产业基地建设。坚持产业基地建设与工业园区建设有机结合，把工业园区作为加快产业集聚的最大项目来做。敏锐抓住东部沿海发达地区产业梯度转移的有利时机，重点推进“有型经济、有根产业、有税企业”的发展，加速实施“111”大产业建设工程，即紧盯纳税1亿元以上企业、投资10亿元以上项目、销售收入100亿元以上产业基地。积极争取国家商务部批准宜春为中西部地区加工贸易梯度转移重点承接地。对已具备一定产业特色的园区，着力择商选资，进一步创特色、上规模、成集群；对综合性园区，根据产业布局、发展空间、区位条件等因素，突出投资大、链条长、后劲足的先进制造业项目。坚持依法依规、集约节约用地，切实保障重大产业和重点项目用地。健全入园项目评价体系，推进产业链式化延伸、企业集群式组合、资源循环式利用。加快园区配套设施建设，增强要素集聚和资源配置功能。按照“引进一个，带来一群；落户一家，聚集一片”的要求，围绕优势产业和龙头企业，发展上下游关联企业。在做强现有“六大产业基地”的基础上，进一步发展机电、食品等特色产业基地，努力扩大产业发展的比较优势和竞争优势。完善信用担保体系，加大异地融资力度，争取外地金融机构落户我市，加快地方商业银行组建，促进有效信贷投入。

　　加紧重大项目建设。继续推进裕盛、匹克、信星鞋业，蓝恒达盐化，斯米克、东鹏、新中源、新明珠、唯美陶瓷，嘉利机电，沃得机械，天合硬质合金，金源纺织，大华无碱玻纤，港元硅业高纯硅，丰城港曲江码头等一批大项目的扩建、投产和落户。加快洪屏电站和明月山机场前期工作，力争年内开工建设。做好丰电公司三期前期工作，认真组织实施一批电力工程新建、扩建和扩容项目。宜春至安福、320国道万大一级公路完工并验收，完成高安至胡家坊一级公路、丰城剑邑大桥续建工程，启动樟树至胡家坊一级公路改造工程，争取105国道丰城至樟树段、丰城至乐安一级公路项目开工建设。加快修建一批旅游公路。

　　二、统筹城乡建设抓协调，加快城镇化进程新步伐

　　以加强新农村建设为重点，提升“三农”工作水平。按照“三清、三改、三配套”的要求，突出抓好新农村建设试点自然村村庄整治工作，继续抓好交通沿线村镇环境整治。大力实施“乡村清洁工程”，逐步实现“走平坦路、喝干净水、上卫生厕、用洁净能源、住整洁房屋、建绿色村庄”的目标。深入开展农村和谐文化建设“一村一品”活动，做到村民活动有场所、娱乐有项目、学习有资料、健身有场地，在有条件的村建立“农家书屋”。重点支持优质水稻、生猪、家禽、肉牛、水产品、油茶、中药材、棉麻、特色蔬菜水果、有机农产品十大主导产业发展，继续实施农业产业化“十百千”工程，提高农产品商品率和附加值，确保农产品加工工业产值占农业总产值的比重比上年提高2个百分点以上，主要农产品转化率达到50%以上。积极发展“一村一品”，全市主抓100个“一村一品”专业村，市本级主抓10个示范点。大力发展农民专业合作经济组织，重点扶持100家市级农民专业合作社示范点建设。大力推进“造地增粮富民”工程，加强耕地保护，稳定粮食生产，抓好江西天地粮食集团建设，推进粮食产业化经营。抓好重大动物疫病防治，确保畜牧业生产安全。加快农村公路、农村客运网络和县乡公路交通安全设施建设，新建水泥（油）路1270公里，新增通水泥（油）路的建制村260个，实现建制村通畅率81％。继续加强水利基础设施建设，全面完成列入省二期病险水库规划的小（一）型、小（二）型水库除险加固，加快丰城鸦丰联圩、奉新宋埠堤等圩堤整治进度，切实抓好农村饮水安全、灌区节水改造、小型农田水利、水电电气化县、小水电代燃料等工程项目建设。加强农村沼气建设，年内建设1万个户用沼气池，并在规模养殖场大力推行环保沼气工程。推进“万村千乡市场工程”和“信福工程”，加快赣西农副产品批发市场扩建。

　　以决胜“三大战役”为抓手，促进城市迅速扩张。加速“三大战役”向全市纵深推进。完善宜春经济开发区封闭运行体制，凝聚各方力量招商，促进产业聚集壮大，努力建成市本级经济增长极和全市园区经济龙头。依托各县市工业园区，培育骨干企业，壮大支柱产业。按照繁荣城市经济、构建“一核、三轴、五组团”城市发展框架的策略，加快实施中心城区人口五年倍增计划。突出抓好宜阳新区重点工程建设，加速推进袁州新城建设，完善各县市城市基础设施，增强城市功能，提升城市品位和形象。加强月亮文化的打造与推介，精心筹办第二届月亮文化节暨经贸活动周，加快配套项目建设。进一步完善全市红色文化、绿色山水、生态农业、古色文化、禅宗文化五条精品旅游线路，打造A级景区群。加强旅游基础设施建设，加大宣传营销力度，组建宜春旅游集团，促进旅游产业发展，力争全市接待海外游客3.45万人次，实现外汇收入858万美元以上；接待国内旅游者720万人次，实现综合收入36亿元以上。抓紧城镇体系规划和县市土地利用规划的编制，推进以宜春中心城区为中心、以县市城区为次中心、以乡村集镇为节点的城镇群建设。全面启动第二次全国土地调查。

　　以完善配套设施为基础，优化城市功能。加快实施城南排水二期工程，继续抓好城市防洪工程建设，开工建设中心城区第三级拦河橡胶坝工程。完善供水网络，消除城市低压供水片区，全面推行“一户一表”改造。积极稳妥完成市供气公司改制改气工作，让市民尽早用上安全、清洁的天然气。继续实施道路通畅工程，启动宜春南路、明月南大道开通工程，完成秀江东路改造工程，拓宽改造宜春大桥，新建明月大桥;实施高士北路延伸工程，实现与宜春经济开发区金发大道的对接；进一步抓好小街小巷和“城中村”改造。认真落实城市公交优先发展政策，合理整合城乡公交资源，逐步实现城乡公交一体化。尽快启动宜春经济开发区污水处理和市污水处理厂续建工程；丰城一期日处理3万吨城市污水处理工程力争年内完工；樟树一期日处理4万吨以及高安一期城市污水处理工程争取全面开工建设；加快奉新、上高、宜丰、铜鼓、万载等地的城市污水处理工程前期工作。加快县市垃圾处理场建设。

　　三、突出改革开放抓拓展，激发经济快速增长新动力

　　强力推进招商引资。深入开展 “重大产业招商年”活动，实施“1181”计划，即：精心包装100个重大招商项目，组织100场经贸洽谈会，力争引进投资1000万美元或亿元人民币以上重大项目80个，超5亿元人民币重大项目10个以上。大力开展以商招商、驻点招商、基地招商等活动，加速提升产业聚集规模。加强调度督查，强化跟踪推进，提高项目的履约率、进资率、开工率和投产率。加快转变外贸增长方式，扶持出口大户和高新技术、自主知识产权、自主品牌产品出口；完善口岸服务体系建设，提升“大通关”效率。继续发挥宜春海程公司“领头羊”作用，鼓励和引导有条件的企业拓展外经合作。增强跑项争资实效，按照“研究政策谋项目、明确方向选项目、强化责任争项目”的要求，在分析研究国家产业政策和投资方向的基础上，精心做好项目的运作开发及前期准备，优化和充实项目库。及时掌握信息，加强跟踪联系，特别要密切

与国家、省有关部门的联系沟通，提高跑项争资的针对性、有效性，在争取更多国家和省级项目落户我市上取得突破。

　　积极推动企业上市。重点围绕特色产业基地和支柱产业，大力培养符合产业政策、具有发展潜力的高新技术企业、地方骨干企业和行业龙头企业，加快推进企业上市进程。对拟上市企业坚持做到改制储备一批，辅导申报一批，上市发行一批，做大做强一批；对上市基础较好的企业实施政策扶持，加强跟踪服务，积极做好上市前期工作，争取尽快进入辅导。认真研究和探索“借壳”上市、挂靠上市等途径，引导企业主动靠拢上市公司，早日进入资本市场。力争年内华伍起重、江西润田、南特公司、百神医药等企业完成上市准备。

　　深化经济领域各项改革。完善国有资产监督管理工作。抓紧启动宜春汽运公司改制工作，积极稳妥推进井竹麻业、三九宜工生化的重组，加紧做好长青机械责任有限公司企业破产改制工作。深化农村综合改革，重点推进乡镇机构改革，稳步实施国有农场税费改革，切实抓好林业配套改革，认真落实各项惠农政策，严防农民负担反弹。积极推进小型水利工程产权制度改革，加快农村用水协会建设。继续深化部门预算改革、国库集中收付制度改革，全面推进“乡财县代管”，启动公务卡改革试点。继续加快推进事业单位改革，推行市直事业单位全员聘用制，完善事业单位社会保障体系。

　　加大自主创新引导力度。积极开展科技对外合作与交流，大力引进和培养科技人才，鼓励和引导企业以自研、引进、联合开发等方式进行创新，不断提升全市自主创新能力和科技实力。深入推进科技入园工程，加快宜春经济开发区科技孵化基地建设。大力实施科技进村入户工程，继续从高校、科研所和市县涉农部门选派1000名科技特派员进村，引导和带动更多的农业技术人员深入农村一线开展技术服务。切实抓好知识产权保护，建立健全知识产权保护体系，鼓励和引导企业、高校、科研机构和科技人员开发、申请专利技术，全市专利申请量年增长10%以上。

　　四、优化环境品牌抓创建，实现对外影响力新提升

　　进一步优化生态环境。良好的生态环境是我市的最大品牌和第一名片。坚持生态立市、绿色发展战略，严格实施生态市规划，严把建设项目环保准入关，严格执行节能减排目标责任制度和环境影响评价制度。深入开展工业园区污水治理、中心城区饮用水源保护、中心城区有毒有害气体零排放、重点流域水污染整治等专项行动。正式启动宜春市污染源自动在线监控系统的建设，全面完成全市污染源普查工作。逐步淘汰能耗高、污染重、技术落后的企业，重点在煤炭、竹木、化工、建材等行业中开展资源循环利用。加强水源涵养、水土保持等重要生态功能区的保护和建设，抓好造林绿化，提高森林覆盖率，加强生态公益林、天然阔叶林保护，加大治理水土流失力度。全面治理农村水源污染，力争年内建成一批生态村试点和国家级环境优美乡镇试点。继续开展玉京山升格省级自然保护区、靖安九岭山升格国家级自然保护区的申报工作，争取年内申报成功。

　　进一步优化宜居环境。积极推进“最佳宜居城市”建设，在中心城区着力抓好宜居的硬环境和硬条件建设。硬环境建设即实施“13579”绿色工程，今年完成10万株树木种植任务，开工建设9个绿色文化广场、2条景观大道、15个游园及滨江公园，提质改造袁山公园和状元洲公园，积极开展“公益林基地创建活动，力争完成动物园、花鸟园、盆景园建设和体育广场改造。硬条件建设即实施“五个一”便民工程，用三年左右时间，新建一批劳动就业服务平台、一批中小学校和幼儿园、一批医疗卫生机构、一批农贸和百货市场、一批社区公共服务场所，解决市民日常生活便利和便宜的需求。进一步巩固和提升中心城区城市创建各项成果，完成“创卫”迎检工作并健全“保卫”长效机制；加强县市创建省级以上园林城、卫生城等工作，力争各有2个县市申报并命名。进一步健全城市管理科学方法和长效机制，深化市政环卫、绿化综合管护改革，加强城市规划建设全程监管，严厉查处违法违章建筑，规范房地产市场开发，始终保持优美的城市环境。

　　进一步优化创业环境。围绕打造“诚信宜春”形象品牌，积极构建政府、企业和个人信用体系，营造最适宜企业成长的“生态”环境。按照“归并职能、整合科室、集中办理、充分授权”的要求，深入推进行政审批制度“两集中、两到位”改革。抓好行政服务中心建设，逐步推行网上申报、网上受理、网上审批。严格入企检查准入，健全投诉受理、处理、督查和举报奖励制度，形成快查直处反应机制，真正做到“有报必查、有查必果、查必到人、查必到位”。大力培育创业文化，建立有效推进机制，落实各项政策措施，激活各类创业主体，着力支持经营管理人员和科技人员领头创业，吸引外出务工经商人员返乡创业，扶持下岗失业人员自主创业，鼓励高校毕业生自立创业，推动广大农民致富创业。进一步放宽市场准入，健全社会服务体系，让更多创业者拥有施展才华的平台，使更多民营企业发展壮大。积极扶持“高安货运大军”、“丰城家具大军”、“樟树保险柜大军”、“靖安漂流大军”，打响宜春创业品牌。加强对民营企业家和企业经营管理人才的培养。继续对纳税“百强民营企业”、“客商创业20佳”、“本土创业20佳”进行大张旗鼓的表彰奖励。全市民营经济实现增加值、营业收入均增长30%，实现税收增长35%。

　　五、坚持以人为本抓发展，构建和谐社会新局面

　　继续推进民生工程。第一，切实做好就业和再就业工作。加大财政对促进城乡就业再就业资金投入，建立和完善市、县两级创业指导中心和街道、乡镇创业指导平台，扩大创业培训覆盖面。充分发挥小额担保贷款促进就业的作用，确保小额担保贷款规模达到1.5亿元。建立健全促进“零就业家庭”就业的长效机制，使有就业愿望和就业能力的“零就业家庭”至少有一人就业。推进农村劳动力就近就地转移，确保工业园区和企业与劳动者签订就业招聘合同、与培训机构签订定向培训合同覆盖面达100%，劳动力资源台帐、工业园区用工台帐、培训机构管理台帐达100%。第二，切实完善社会保障。突出抓好社会保险扩面征缴，加大破产改制企业社会保险欠费清欠力度，确保养老、医疗、失业、工伤和生育保险扩面和基金征缴目标任务的完成。做好企业退休人员养老金待遇调整工作，确保企业离退休人员养老金按时足额发放，社会化发放率和支付率达到100%。全面推进城镇居民医疗保险，重点做好低保对象和残疾人员的参保。继续做好国有农、林、水困难企业职工参加医疗保险工作，稳步推进进城务工农民参加养老保险，逐步使农民工享受与城镇从业人员同等社会保障待遇。第三，切实完善生活保障。加强社会救助、社会福利、慈善事业和农村养老保险工作，认真做好农村低保扩面提标工作，抓好各项救助制度实施。坚持应保尽保、动态管理，积极做好与再就业、社会保险、最低工资制度的衔接，不断提高保障水平。加强残疾人、儿童福利设施建设，给他们更多的生活和健康关爱。加强农村敬老院、社会福利院和光荣院建设与管理，全市农村“五保”对象集中供养率达到80%以上。完善流浪乞讨人员救助制度，加快未成年人救助中心建设，切实保护流浪儿童的合法权益。继续加快廉租住房和经济适用住房建设，确保中心城区完成建设8万平方米、约1000套经济适用住房；廉租住房覆盖面从人均住房使用面积7平方米以下扩大到8平方米以下，以租赁补贴为主，新建、购置一批廉租住房提供给城市低收入家庭住房困难户。第四，切实完善教育保障。全面实行城乡义务教育免学杂费和教科书费，提高义务教育阶段公办中小学公用经费标准，健全非义务教育阶段特殊困难家庭学生资助政策体系。认真实施农村中小学校舍维修改造，积极化解农村义务教育债务。将农民工子女义务教育纳入流入地政府教育发展规划，以全日制公办中小学校接收为主，享受与城镇居民子女同等待遇。

　　加快社会事业发展。巩固提高义务教育，促进义务教育均衡发展，重视学前教育，关注特殊教育，加快发展高中教育、高等教育和成人教育。鼓励和扶持社会力量办学，优化高中阶段教育结构，高中阶段教育毛入学率达到70%以上。大力发展职业教育，力争有2-3所中等职业学校进入国家级重点职校行列，做大做强宜春学院，努力提升办学内涵，做好宜春职业技术学院迎接教育部高职高专院校人才培养工作水平评估。进一步实施人才强市战略，着力深化人事制度改革，着力加强人才队伍建设，着力抓好各项人事法规制度落实，着力构建人事公共服务体系。积极开展技术标准企业试点，加快标准化工作步伐。继续做好靖安、铜鼓、高安等地国家级农业标准化示范区建设和验收工作。大力实施名牌带动战略，争创中国名牌产品1个 。完善公共卫生医疗救治体系。加强农村中医卫生工作。加强农村医疗网点建设，改善农村医疗卫生服务，不断提高医疗卫生水平，继续深入推进食品药品安全综合监管体系建设，切实维护公众饮食用药安全。加强社会主义精神文明建设，努力提高公民道德素质。推进公共文化服务体系和文化设施建设，完善市图书馆相关设施，启动市博物馆新馆和市科技馆建设。重视文艺精品创作，加强文艺人才培养。在抓好专业文艺团体改革创新的同时，积极支持民营文艺表演团体的发展。充分发挥各种文艺协会的作用，促进文化事业繁荣。加大文物保护工作力度，重点抓好第三次全国文物普查工作，做好靖安东周古墓申报国家级文物保护单位前期工作，争取铜鼓秋收起义前敌指挥部旧址—肖家祠成功申报。广泛开展全民健身运动，提高竞技体育水平，争取承办更多的大型体育赛事，进一步扩大宜春对外影响。认真开展人口和计划生育“落实年”活动，重点抓好党员干部和社会公众人物违法生育查处、农村和城镇流动人口管理，切实落实以六项工程为主要内容的奖励扶助措施，进一步加强出生人口性别综合整治力度，促进全市人口和计划生育工作上新水平。

　　高度重视安全稳定。强化安全监管主体责任，全面落实安全生产责任制，加强和改进安全基础建设。着力抓好重点行业安全，严格整治规范煤矿开采，加快烟花爆竹结构调整，坚决打击非法生产、非法采矿行为，确保全市安全生产形势稳定。继续深入开展“和谐平安校园”创建活动，切实加强学校安全工作。完善群众利益诉求表达机制和利益矛盾调处机制，加强市、县两级接访中心和信访信息化建设，进一步发挥市人民来访接待中心的功能，抓好市长专线、市长信箱建设，重视人民建议征集工作，拓宽群众信访渠道。健全重大突发事件的预警应急机制，增强对自然灾害、事故灾难、突发公共卫生和公共安全事件的预防和处置能力。加强安全稳定基层基础工作，健全社会治安防控体系，推进社会治安综合治理，深入开展平安创建活动，改革和加强社区警务工作，依法防范和打击各类刑事犯罪活动，增强人民群众的安全感。重视国家安全工作。重视国防教育，提高民兵预备役建设质量，搞好国防动员，扎实做好军事斗争准备。完善村民自治，组织好村委会换届选举。

　　各位代表，全面完成今年各项目标任务，实现全市经济又好又快发展，各级政府和政府各部门责任重大、使命光荣。面对新的形势和繁重任务，必须切实加强政府自身建设，致力提高领导和组织科学发展的能力。

　　第一，加强学习创新，在提升软实力中有更大作为。实现我市发展的新跨越，最要紧的是实现软实力的新超越。我们要自觉而始终围绕观念、战略、诚信、效率、创业文化、人才等关键内涵，不断加强学习，切实转变观念，特别注重战略思维，坚持正确的发展定位和取向，坚定不移地以诚信和效率优化投资创业环境，坚定不移地弘扬创业精神，坚定不移地更加注重人才、培养人才、用好人才。各级政府领导干部务必将提高软实力、优化投资创业环境作为推动经济社会又好又快发展的关键举措来抓，思想上高度重视，工作中找准切入点，坚持身体力行，当好表率，进一步打造宜春软实力新优势，促进宜春经济社会跨越式发展。

　　第二，规范权力运行，在服务发展大局中有更大作为。坚持依法行政、廉洁从政，理顺行政执法体制，合理划分上下级行政机关执法权限，适当下移执法重心；在巩固中心城综合执法改革试点的基础上，推动综合执法向相对集中行政处罚权过渡，并向基层延伸；坚持管理与服务并重、教育比处罚先行、执法与疏导结合，实行文明执法、人性化执法。加强执法监察、廉政监察和效能监察，加大对重点领域、重点部门、重点资金、重点项目的审计力度，不断健全经济责任审计制度。坚持预防与惩治相结合，进一步加大从源头上防治腐败力度。全面推行政府信息公开，确保政府工作透明运行。主动接受市人大及其常委会的监督，自觉接受市政协民主监督，认真听取民主党派、工商联、无党派人士和各人民团体的意见，高度重视社会公众监督和新闻舆论监督。

　　第三，创新工作模式，在破解发展难题中有更大作为。坚持用创新的思路谋划工作，用创新的实践破解难题，用创新的办法创造机遇。各级政府及其工作人员，要在完成常规动作基础上，善于拿出自选动作，敢于尝试高难动作，推陈出新，精益求精，不断创造更多特色和亮点。要围绕解决制约发展的突出问题，充分发挥主观能动性，善于研究，精于运作，突破资源依赖、区域限制和既有模式的局限，实现无中生有、小中求大，拓展发展新领域，培育发展新优势，勇当破解难题的尖兵，不断开创新局面。

　　第四，加大执行力度，在落实发展思路中有更大作为。各级政府要牢固树立“全市一盘棋”思想，令行禁止，服从大局，市委、市政府任何工作只要部署下去，就要不折不扣地执行到位。政府领导干部要牢记重托，以落实为天职，以得失论成败，指挥靠前，操作至上，在有限的时间里，创造出经得起各方检验的业绩。政府全体工作人员，都要大兴调研之风，吃透上情、摸清下情，发现薄弱点、找准着力点，把各项工作做精、做细、做到位。要实行工作推进项目化、项目落实责任化，建立纵向到底、横向到边的落实机制。对每一项工作尤其是重要工作、重点工程、重大项目，都要定人员、定责任、定时间、定进度，建立主管领导催办、责任领导主办、责任部门承办、专门机构督办的督查机制，严格考核，奖惩分明。

　　各位代表！新的篇章令人鼓舞，新的号角催人奋进。让我们紧密团结在以胡锦涛同志为总书记的党中央周围，在省委、省政府和市委的坚强领导下，始终保持敢为人先的创新精神、不进则退的忧患意识、求真务实的拼搏作风，抢抓机遇，扎实工作，为全面建设和谐小康宜春而努力奋斗！
